# Early Biomarkers, Risk Factors, and Functional Indicators of Healthy Longevity and Their Relationship with Diet

**DOI:** 10.3390/nu18111664

**Published:** 2026-05-22

**Authors:** Daniela Martini, Mariangela Rondanelli, Lorenzo Morelli, Francesco Landi

**Affiliations:** 1 Division of Human Nutrition, Department of Food, Environmental and Nutritional Sciences (DeFENS), Università Degli Studi di Milano, 20133 Milano, Italy; 2 Fondazione Istituto Danone, 20159 Milano, Italy; 3 Department of Public Health, Experimental and Forensic Medicine, University of Pavia, 27100 Pavia, Italy; 4 Department for Sustainable Food Process-DiSTAS, Università Cattolica del Sacro Cuore, 26100 Cremona, Italy; 5 Fondazione Policlinico Universitario Agostino Gemelli IRCCS, 00168 Rome, Italy; 6 Department of Geriatrics, Orthopedics and Rheumatology, Università Cattolica del Sacro Cuore, 00168 Rome, Italy

**Keywords:** longevity, aging, health, markers, diet, nutrition

## Abstract

**Background/Objectives:** Healthy longevity depends on not only lifespan but also the maintenance of physiological, metabolic, physical, and cognitive functions throughout aging. Identifying early determinants of health is crucial for preventing age-related decline. This narrative review aims to synthesize current evidence on how diet and specific nutrients relate to these early risk factors and indicators of healthy longevity. **Methods:** A review was performed to identify the links between dietary factors, energy balance, and gut microbiota composition and normal body weight; blood cholesterol, pressure, and glucose; healthy sleep; an active lifestyle; and normal physical function and cognitive performance. Particular attention was given to Mediterranean and other plant-based dietary models as sources of key nutrients. Evidence from observational studies, randomized controlled trials, and meta-analyses was considered. **Results:** Across all markers, dietary quality and nutrient adequacy emerged as consistent determinants of health outcomes. Key nutrients were associated with favorable cardiometabolic, cognitive, and musculoskeletal functions, such as omega-3 fatty acids, fiber, vitamins D and B, minerals like magnesium and potassium, and polyphenols. Common nutrition gaps included insufficient intake of fiber, unsaturated fats, and micronutrients, which was often linked to a shift toward less plant-based diets. Gut microbiota diversity may mediate several of these associations, influencing metabolism, inflammation, sleep quality, and cognitive performance, although inter-individual variability and causal pathways remain incompletely understood. **Conclusions:** An integrated dietary approach emphasizing the consumption of whole and plant-rich foods, with moderate amounts of animal foods, supports multiple early markers, risk factors, and indicators of healthy longevity. The modulation of the gut microbiota through plant-based diets and fermented foods represents a promising strategy for maintaining health across aging trajectories.

## 1. Introduction

Longevity is not only the result of genetic inheritance, but it also reflects the complex interplay between environmental, biological, and behavioral factors accumulated across the life course. Among these, diet arises as a crucial and modifiable determinant of health lifespan and life expectancy [1]. However, although the association between diet and healthy aging is widely recognized, the strength and consistency of evidence vary substantially across outcomes, populations, and study designs. Disentangling the contribution of specific nutrients or foods from that of overall dietary patterns remains challenging. Recent decades have seen a paradigm shift from focusing only on disease treatment to identifying early determinants that can predict and promote healthy longevity. These include risk factors such as normal body weight and composition, healthy lipid and glucose profiles, and blood pressure control, as well as functional indicators such as adequate sleep, cognitive and physical performance, and an active lifestyle, which are increasingly recognized as not only indicators of current health but also predictors of resilience against age-related decline [2]. At the same time, these determinants are heterogeneous in nature and are supported by different levels of evidence, ranging from observational associations to stronger evidence from intervention studies for selected outcomes. While these parameters are not biological biomarkers of aging per se, they represent modifiable clinical and functional determinants associated with favorable aging trajectories. Notably, each of these markers can be strongly influenced by nutritional patterns and habits. Nevertheless, the available literature is not always consistent, and causal relationships are often difficult to establish because of confounding factors, differences in dietary assessment, and variability in study populations. In this regard, it is important to highlight that the role of diet goes beyond simple caloric balance. It determines and regulates the body composition, controls metabolic and cardiovascular risk factors, and contributes to preserving musculoskeletal and cognitive function. Finally, diet interacts with the gut microbiota, which acts as an important mediator increasingly seen as central in the diet–health–longevity axis.

This narrative review aims to synthesize current evidence on how diet and specific nutrients relate to these early determinants of health. Specifically, this review aims to (i) examine the associations between dietary patterns (with a focus on the Mediterranean diet and other plant-based dietary models), the intake of nutrients and bioactive compounds, and key clinical and functional determinants of healthy aging; (ii) discuss the biological mechanisms that may link dietary exposures to aging trajectories, including metabolic and microbiota-related pathways; and (iii) critically appraise current evidence by highlighting its consistency, limitations, and remaining knowledge gaps, thus supporting the need for further studies integrating traditional clinical indicators with emerging biological markers of aging. By linking diet to measurable early clinical and functional indicators, our goal is to provide an integrated perspective for clinicians, researchers, and public health professionals to identify actionable strategies that foster healthy longevity.

## 2. Materials and Methods

This narrative review aims to provide an integrative overview of early biomarkers, risk factors, and functional indicators associated with healthy longevity and to examine their potential modulation by dietary exposures.

A literature search was conducted using PubMed/MEDLINE (http://www.ncbi.nlm.nih.gov/pubmed, accessed on 18 May 2026), Web of Science (www.webofscience.com), and Scopus (www.scopus.com) up to November 2025, with a primary focus on studies published in the past 10–15 years while including earlier studies where relevant. The search strategy included combinations of keywords and MeSH terms such as “healthy longevity”, “healthy aging”, “biological aging”, “early biomarkers”, “epigenetic clocks”, “inflammation”, “metabolic markers”, “diet”, “nutrition”, and “dietary patterns”.

Studies were selected based on their conceptual relevance to early risk factors and biological indicators predictive of healthy aging trajectories and their association with dietary factors. Priority was given to evidence from large observational cohorts, randomized controlled trials, meta-analyses, and key mechanistic studies. Only articles published in English were included.

The reference lists of the selected papers were also manually screened to identify additional relevant publications. Evidence was synthesized narratively, with attention to the consistency of findings across studies, the strength and limitations of different study designs, and the biological plausibility of the proposed mechanisms.

Given the narrative nature of this review, no formal systematic protocol or quantitative synthesis was applied.

## 3. Results

**Figure 1:**
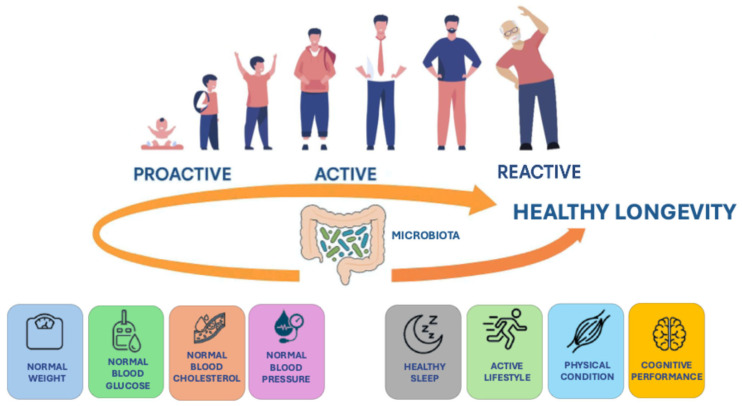
The early risk factors and indicators of healthy longevity.

The early risk factors and indicators of healthy longevity identified in this review are summarized in Figure 1. The following sections examine each biomarker individually, highlighting its physiological significance and summarizing current evidence regarding key nutrients and potential nutrient gaps that may influence its regulation.

### 3.1. Normal Body Weight and Other Anthropometric Measures

#### 3.1.1. Background

The prevalence of inadequate body weight significantly varies across different countries. This variation may depend on several factors, such as socioeconomic status, level of education, and age. Overall, from 1990 to 2022, the age-standardized prevalence of thinness for males and females decreased in 22% and 40% of countries, respectively, while that of obesity increased in 93% and 98% [3].

Maintaining an adequate body weight and body mass index is essential for overall good health, while inadequacies in these parameters are associated with adverse health outcomes throughout the life course. In particular, obesity represents a major global health challenge with important clinical implications, and it is responsible for many deaths and disability-adjusted life years (DALYs) resulting from chronic diseases such as diabetes mellitus, ischemic heart disease, hypertensive heart disease, chronic kidney disease, and stroke [4]. In recent decades, the global deaths and DALYs attributable to high body mass index (BMI) increased more than 2.5-fold. Thus, to address and mitigate the rise in diabetes, cancer, and other chronic diseases, it is essential to target the causes of obesity [5,6] and malnutrition, as indicated by the Sustainable Development Goals [7]. However, it is important to highlight that most of the available evidence linking body weight to long-term health outcomes is derived from observational studies, which may be affected by residual confounding and reverse causality, particularly in older populations. Moreover, the use of BMI as a proxy for adiposity has well-recognized limitations, as it does not account for body composition or fat distribution, which may be more strongly associated with cardiometabolic risk.

#### 3.1.2. Key Nutrients and Nutrient Gaps

Body weight can be affected by several factors, including genotype, age, sex, level of education, and family habits. Lifestyle plays a major role, as obesity is caused by a long-term scenario of energy intake exceeding expenditure. Sustained positive energy balance remains the primary driver of weight gain, with dietary composition influencing satiety, energy density, and metabolic responses within this broader energetic framework. Numerous observational and clinical studies have investigated the role of different dietary patterns on body weight and shown that the Mediterranean diet is associated with the strongest and most consistent evidence of a beneficial effect on anthropometric parameters and on related cardiometabolic risk factors [8]. Nevertheless, much of this evidence originates from observational studies, and the findings from randomized controlled trials, although generally supportive, are sometimes heterogeneous in the magnitude and duration of effect.

Besides the Mediterranean diet, several other dietary patterns also share common features (e.g., plenty of plant-based foods, balance among nutrients) that could be promoted due to their potential role in maintaining or reducing body weight [9]. This means that rather than the role of a single nutrient, food, or diet, current evidence supports a common set of principles that promote health, prevent or manage obesity, and improve overall wellbeing [10]. Among them, a key strategy for weight management that can be leveraged across dietary patterns is to reduce energy density [11]. In this context, dietary fiber, unsaturated fats, and minimally processed plant foods contribute to lower energy density and improved satiety. However, the extent to which these factors independently influence long-term weight regulation remains debated, as dietary behaviors are strongly influenced by environmental and behavioral determinants. Moreover, focusing exclusively on individual nutrients may be misleading if total caloric intake and overall dietary patterns are not considered.

As mentioned above, it is noteworthy that body weight is only one of the several anthropometric measures to be considered for healthy longevity. In particular, visceral adiposity appears to be more strongly associated with cardiometabolic risk than overall body weight, although its measurement is less standardized in large-scale studies. In this regard, in addition to adequate energy intake and exercise, an adequate protein and leucine intake appears to be fundamental for preserving skeletal muscle mass in aging and preventing sarcopenia, which is associated with a higher risk of falls, disability, and death [12]. Finally, it is important to highlight that some studies have reported an “obesity paradox” in older adults, in which higher BMI is not consistently associated with increased mortality, thus highlighting the complexity of interpreting anthropometric measures across different age groups and clinical conditions [13].

### 3.2. Normal Blood Cholesterol

#### 3.2.1. Background

The relationship between diet and longevity is particularly significant for individuals with hypercholesterolemia, especially familial hypercholesterolemia (FH). Lipid markers, together with inflammatory markers, are indicative of healthy aging and longevity.

Each 1 mmol/L (38.7 mg/dL) reduction in LDL-C has been associated with a 22% reduction in major vascular events over a median follow-up of five years when achieved through statin therapy and a 25% reduction in cardiovascular event rates when considering various interventions, including dietary changes [14]. A dietary approach involving a combination of cholesterol-lowering foods (such as plant sterols, soy protein, viscous fibers, and nuts) resulted in a reduction of about 29.6% in LDL cholesterol over the same period [15].

#### 3.2.2. Key Nutrients and Nutrient Gaps

High carbohydrate intake, snack dietary patterns, low fiber consumption, low fat quality, and micronutrient deficiencies are the main nutritional gaps.

Regarding high carbohydrate intake, studies indicate that patients with familial hypercholesterolemia (FH) often consume more carbohydrates compared to control groups. This excessive intake is linked to increased glucose levels and may contribute to lipid metabolism dysfunctions [16]. Moreover, a prevalent dietary pattern identified in various studies is the “snack dietary pattern,” which correlates with a higher risk of hypercholesterolemia. This pattern includes the frequent consumption of high-calorie snacks, thus leading to increased total cholesterol and LDL cholesterol levels [17]. An insufficient intake of dietary fiber is another nutritional gap, particularly that of soluble fibers such as the β-glucans found in oats and barley. These fibers are known to help lower cholesterol levels by binding bile acids and promoting their excretion [18].

Regarding low fat quality, diets high in saturated fatty acids (SFAs) are associated with increased cholesterol levels, while polyunsaturated fatty acids (PUFAs) can lead to significant reductions in total cholesterol and LDL-C levels [19].

In terms of micronutrient deficiencies, patients with hypercholesterolemia may experience deficiencies in essential vitamins (including vitamins D, B6, and B12) and minerals (magnesium, zinc, and selenium) [20]. A significant correlation exists between low levels of vitamin D and increased total and LDL cholesterol (LDL-C) levels. In one study on hyperlipidemic patients, vitamin D deficiency was prevalent, thus indicating its potential role in lipid metabolism regulation [21,22]. Vitamin E has been positively correlated with total cholesterol and LDL-C levels, suggesting that higher vitamin E levels might be associated with worse lipid profiles [21,22]. Additionally, vitamins B6 and B12 have shown inverse correlations with total cholesterol and non-HDL levels in hyperlipidemic patients, indicating that deficiencies may contribute to dyslipidemia [22].

Inadequate magnesium intake has been linked to higher chances of hypercholesterolemia and hypertriglyceridemia, as magnesium appears to be protective against lipid imbalances [23]. Moreover, deficiencies in zinc and selenium have been associated with lower HDL cholesterol levels and may contribute to increased cardiovascular risk factors [23].

Finally, certain branched-chain amino acids (BCAAs) like leucine, isoleucine, and valine have been shown to correlate negatively with HDL levels while being positively associated with LDL and triglyceride levels. This suggests that amino acid metabolism may also influence the lipid profile [24]. However, current evidence remains largely observational, and the mechanistic relationship between BCAA metabolism, cardiometabolic dysfunction, and lipid homeostasis is still being elucidated [25].

### 3.3. Normal Glucose

#### 3.3.1. Background

Individuals with type 2 diabetes at age 50 have shown a reduction in life expectancy of 6 years compared to those without diabetes. However, achieving type 2 diabetes treatment goals may partially mitigate this gap, with the reported increase in life expectancy ranging from 3 to 10 years [26]. Studies show that individuals who maintain good blood glucose control from the onset of diabetes experience fewer complications and a longer lifespan compared to those who do not [27,28,29].

#### 3.3.2. Key Nutrients and Nutrient Gaps

Individuals with diabetes often face significant nutritional gaps that can affect their overall health and management of the condition. These gaps arise from various dietary deficiencies, lifestyle choices, and the specific nutritional needs associated with diabetes. Specifically, diabetic subjects may suffer from micronutrient deficiencies and experience barriers to nutritional adherence due to a lack of information and socioeconomic factors. In fact, many patients report insufficient knowledge about what constitutes an adequate diet [29]. Moreover, low-income individuals may struggle to access healthy food options, thus compounding nutritional gaps [28].

Diabetics are frequently at risk of micronutrient deficiencies due to low food intake and suboptimal dietary patterns [30]. This can lead to a widening gap between nutrient intake and requirements, which is particularly concerning given that nutrient needs may be higher in individuals with diabetes compared to healthy individuals [31]. Common deficiencies include those in magnesium, zinc, and vitamins B12 and D.

In terms of magnesium, a logistic regression analysis found that higher serum magnesium levels significantly reduced the odds of poor glycemic control [32]. A recent study performing pooled analyses of 24 randomized controlled trials with 1325 T2D individuals reported that a daily magnesium supplementation of 250 mg significantly improved HbA1c from 8.32% to 7.96% over three months. Dose–response analysis suggested that a daily intake of 279, 429, and 300 mg for 116, 88, and 120 days may be associated with improvements in glycemic control, lipid profile, and blood pressure, respectively [33].

Meanwhile, zinc activates pathways that lead to the phosphorylation of proteins, such as Akt and ERK1/2, which are critical for maintaining glucose homeostasis [34]. One study found that a significant percentage of type 2 diabetic patients had dietary intakes below the estimated average requirement for zinc [35]. This inadequacy is exacerbated by dietary patterns that are often low in meat, fish, and dairy products, which are key sources of zinc [36].

Another study found that approximately 12.9% of diabetic patients had vitamin B12 deficiency, with a significant proportion (69.8%) of these patients being treated with metformin [37]. Metformin is known to interfere with vitamin B12 absorption, leading to lower serum levels over time, as recently reported in a review by Kibirige [38]. Some studies have suggested that addressing vitamin B12 deficiency could improve metabolic health and potentially enhance glucose handling [38,39]. The regular monitoring of vitamin B12 levels in diabetic patients, especially those on metformin, may be advisable to prevent deficiency-related complications.

A randomized controlled trial reported that individuals receiving vitamin D had a significantly lower Homeostatic Model Assessment of Insulin Resistance (HOMA-IR) score compared to a placebo group, indicating better insulin sensitivity [40]. However, these findings are derived from specific study populations and may not be generalizable. Additionally, progression towards diabetes was markedly reduced in the vitamin D group (3% vs. 28% in the placebo group) [40], although this estimate is based on a single study and should be interpreted with caution. More broadly, evidence on the role of vitamin D supplementation in diabetes prevention remains heterogeneous. For example, the D2d trial did not demonstrate a statistically significant reduction in diabetes incidence with vitamin D supplementation in the overall population, although potential benefits were observed in selected subgroups.

Similarly, meta-analyses have suggested a modest reduction of 10% to 13% in diabetes risk among individuals with prediabetes, but results vary across studies and are influenced by differences in study design, baseline vitamin D status, and the use of supplements [41,42]. Observational studies have consistently reported that higher circulating levels of 25-hydroxyvitamin D are associated with a lower incidence of type 2 diabetes; however, these associations do not establish causality and may be affected by residual confounding and reverse causation [43].

Finally, we report that the Endocrine Society’s recent Clinical Practice Guidelines on Vitamin D for the Prevention of Disease suggest empiric vitamin D supplementation for people with high-risk prediabetes [24,44].

In conclusion, lifestyle modification must be a routine management component for adults with diabetes, and research highlights several dietary patterns that are associated with increased life expectancy in diabetic patients. Adhering to recommendations of a diet rich in whole grains, fruits, vegetables, and nuts and low in processed foods has been linked to substantial gains in life expectancy in diabetic patients. Moreover, people with diabetes are frequently at risk of micronutrient deficiencies due to low food intake and suboptimal dietary patterns. This can lead to a widening gap between nutrient intake and requirements. Common deficiencies include those in magnesium, zinc, and some vitamins, such as vitamins B12 and D. These nutrients appear to play a crucial role in modulating insulin sensitivity and reducing the risk of type 2 diabetes, particularly among those with prediabetes. Finally, several limitations should be acknowledged. Variability in genetic background, socioeconomic status, dietary habits, and lifestyle, as well as study design and sample size, may influence the observed associations.

### 3.4. Normal Blood Pressure

#### 3.4.1. Background

The interplay between early markers of hypertension and dietary habits underscores the importance of lifestyle modifications for promoting healthy aging. The effective management of blood pressure through dietary interventions can lead to improved longevity outcomes by mitigating risks associated with cognitive decline and cardiovascular diseases [45].

#### 3.4.2. Key Nutrients and Nutrient Gaps

Addressing nutritional gaps in hypertensive patients involves a comprehensive approach that includes promoting diets rich in potassium, calcium, magnesium, and fiber and low in sodium. The DASH and Mediterranean diets are particularly effective for managing hypertension [46]. Overcoming barriers related to adherence and cultural preferences is essential for successful dietary interventions. In terms of essential nutrients, potassium, calcium, and magnesium are the most important minerals involved, and they play a vital role in blood pressure regulation [47]. Increased potassium intake, particularly from fruits and vegetables, helps mitigate sodium’s effects and promotes vasodilation, which can lower blood pressure [48,49]. Calcium and magnesium also contribute positively, but their supplementation remains controversial due to inconsistent evidence regarding their direct impact on hypertension management [49].

A recent meta-analysis of 38 RCTs supports the beneficial effect of magnesium on reducing BP among populations with hypertension and hypomagnesemia, although effects should be interpreted with caution due to the high heterogeneity of studies [45].

Limiting sodium intake is fundamental in controlling hypertension. A reduction to less than 5 g daily can lead to significant decreases in both systolic and diastolic blood pressure [50].

Considering the adherence issue, patients often struggle with maintaining dietary changes due to lifestyle habits, preferences, and socioeconomic factors [48]. Regarding cultural preferences, dietary recommendations may clash with cultural food practices, thus making it difficult for patients to adopt suggested diets without feeling deprived or alienated [48]. Moreover, research indicates that many individuals with hypertension often have poor nutritional knowledge and unhealthy dietary habits. One study found that hypertensive patients had a higher body mass index (BMI) and a lower health-promoting diet index compared to non-hypertensive individuals [51]. This suggests that addressing nutritional education and dietary choices is crucial in managing hypertension. Thus, the role of healthcare providers is critical in educating patients about the importance of nutrition in managing hypertension. Continuous support can improve adherence to dietary recommendations [48].

In conclusion, the interplay between early markers of hypertension and dietary habits underscores the importance of lifestyle modifications for promoting healthy aging. The effective management of blood pressure through dietary interventions can lead to improved longevity outcomes by mitigating risks associated with cognitive decline and cardiovascular diseases. Moreover, understanding the nutritional gaps in hypertensive patients is crucial for effective management and prevention strategies. Addressing nutritional gaps in hypertensive patients involves a comprehensive approach that includes promoting diets rich in potassium, calcium, magnesium, and fiber and low in sodium, and larger, well-designed studies assessing the dose–response relationship between nutrient intake and BP and identifying potential optimal supplementation strategies for subpopulations may be necessary.

### 3.5. Healthy Sleep

#### 3.5.1. Background

Adequate sleep is a fundamental biological process required for the survival and development of humans. It is an essential condition for ensuring an adequate quality of life [52], affects both academic and occupational performance and efficiency [53,54], and allows overall health to be maintained. Multiple dimensions of sleep, including duration, quality, and timing, have been associated with a broad range of health outcomes. These consist of cognitive, psychosocial, and cardiometabolic conditions, including type 2 diabetes, cardiovascular disease, stroke, and obesity [55,56,57]. For all these reasons, insufficient and/or low-quality sleep represents a serious challenge in modern societies worldwide, commonly affecting a large segment of the population, including adolescents [58], shift-workers [59], and community-dwelling older adults [60].

#### 3.5.2. Key Nutrients and Nutrient Gaps

A healthy diet and adequate sleep are linked in a bidirectional manner. On the one hand, habitual sleep duration has been associated with higher food and calorie intake [61,62] and absolute or relative intake of nutrients or foods [63].

On the other hand, there is growing scientific evidence suggesting that the consumption of specific foods may positively or negatively affect sleep. In particular, the consumption of healthy foods, such as fish, was associated with better sleep quality. In contrast, a higher intake of processed and free-sugar-rich foods, as well as unhealthy dietary habits such as skipping breakfast, was correlated with worse sleep duration and quality [63,64].

Regarding nutrients, it is hypothesized that carbohydrate intake is associated with sleep quality. Studies on the quality of carbohydrates have shown mixed results, while carbohydrate quantity seems to affect sleep quality. Fiber intake has been linked to greater deep sleep, whereas that of sugar has been associated with lighter sleep [64,65]. Thus, the low intake of fiber and the high intake of sugar often observed in many populations may represent risk factors for poor sleep. This further supports the importance of promoting the consumption of fiber-rich foods, such as legumes and whole grains, but the recommendations of different dietary guidelines are often not met, particularly those promoting the Mediterranean diet [66].

Moreover, epidemiological studies have observed that diets higher in protein and lower in saturated fats are associated with better sleep quality, while diets low in omega-3 PUFAs are linked to disturbed nocturnal sleep. These results further support the importance of consuming a higher-quality diet for improving this early marker of healthy longevity [65].

Overall, sleep may influence healthy aging through its effects on metabolic regulation, inflammation, and neurocognitive function. While causal relationships remain incompletely established, adequate sleep appears to support resilience against age-related decline and may contribute to more favorable aging trajectories. At the same time, emerging evidence suggests that dietary factors (e.g., macronutrient composition, micronutrient adequacy, and overall dietary patterns) may modulate sleep characteristics, although the findings remain heterogeneous and partly inconsistent across study designs. In this context, the bidirectional relationship between diet and sleep represents a potentially relevant pathway linking nutritional exposures to healthy aging, although further longitudinal and interventional studies are needed to clarify causality and underlying mechanisms.

### 3.6. Active Lifestyle

#### 3.6.1. Background

Physical activity (PA) programs have beneficial effects on numerous organ systems, affecting the development and advancement of a variety of chronic conditions, including cardiovascular disease, type 2 diabetes, osteoporosis, and certain types of cancer [67,68].

Physical activity exerts its beneficial effects through multiple biological mechanisms. Regular exercise improves mitochondrial function and biogenesis, enhances insulin sensitivity, and reduces chronic low-grade inflammation, which is a key driver of aging and age-related diseases [69]. Furthermore, exercise stimulates muscle protein synthesis through the activation of anabolic pathways, including the mTOR signaling pathway, and helps counteract anabolic resistance commonly observed in older adults. It also promotes neuromuscular function and coordination, contributing to balance and fall prevention. Additionally, physical activity supports vascular health by improving endothelial function and cerebral blood flow, which may partly explain its positive effects on cognitive function and mental health.

Additionally, regular exercise is a well-established determinant for the prevention of sarcopenia and frailty development, contributing to the reduction in fall risk and advancing mobility and independence in older adults [70]. Exercise is also prescribed for the treatment of various painful conditions and has been shown to exert beneficial effects on mental health. It can be viewed as an alternative or complementary approach in treatment strategies for mood disorders and cognitive impairment [71,72].

PA and structured exercise training (ET) differ in intensity and purpose, but both contribute to improved health outcomes. PA, including daily activities such as walking, is associated with reduced all-cause mortality and improved functional status, even at low-to-moderate levels. In contrast, ET, which is planned and structured, may provide additional benefits in terms of cardiorespiratory fitness, muscle strength, and metabolic health. Importantly, evidence suggests that both PA and ET are associated with gains in life expectancy and reductions in disability-adjusted life years (DALYs), with benefits observed across all age groups. Notably, initiating physical activity even in later life is associated with significant improvements in survival and functional capacity, although earlier and sustained engagement yields the greatest benefits.

Conversely to PA, a sedentary lifestyle is associated with increased all-cause mortality, indicating the critical need to include physical activity in daily life to promote healthy aging and increased longevity [73].

#### 3.6.2. Key Nutrients and Nutrient Gaps

Optimal nutrition is essential to support an active lifestyle, especially in older adults, who may have altered metabolic needs, thus bolstering the hypothesis that multicomponent interventions are needed to preserve mobility in vulnerable older people [74]. Protein intake is a key factor in maintaining muscle mass and function [75]. The literature suggests that high protein intake, particularly that of leucine-rich foods, can activate muscle protein synthesis and prevent sarcopenia [76]. Despite this significance, many older adults fail to meet the recommended protein amount, thereby leading to muscle wasting and functional decline [77].

Carbohydrates play a crucial role in energy metabolism, supplying the necessary fuel for physical activity. Complex carbohydrates with a low glycemic index, including whole grains, legumes, and vegetables, are ideal since they can sustain the slow release of energy and promote metabolic health [78]. Proper hydration is also required to maintain endurance and prevent fatigue, yet dehydration is a common issue among older adults due to decreased thirst perception [79].

Micronutrients such as vitamin D, calcium, and magnesium are essential for bone development and muscle function. Vitamin D deficiency is particularly concerning, as it has been linked to a reduction in muscle strength, an increased risk of falling, and a higher rate of bone fractures [80]. Omega-3 fatty acids have also been shown to reduce the amount of exercise-induced inflammation, ensure recovery, and thus play a beneficial role in individuals engaging in regular physical activity [81].

### 3.7. Cognitive Performance

#### 3.7.1. Background

Cognitive performance is a key determinant of healthy longevity, influencing memory, learning, decision-making, and overall wellbeing. Age-related cognitive decline is associated with neurodegenerative disorders such as Alzheimer’s disease (AD) and other dementias, which significantly impact quality of life and independence in older adults [75]. While genetic predisposition is a participant in cognitive aging, lifestyle determinants like nutrition and diet have emerged as crucial modifiers of brain function [82,83]. Nutritional deficiencies, oxidative stress, and chronic inflammation are among the leading contributors of cognitive impairment and highlight the importance of a well-balanced diet as being essential for preserving cognitive function [84]. However, the strength of evidence supporting the role of diet in cognitive aging varies across study designs, and causal relationships remain difficult to establish.

#### 3.7.2. Key Nutrients and Nutrient Gaps

Several nutrients are essential for maintaining cognitive performance and reducing neurodegenerative disease risk. Omega-3 fatty acids, in particular docosahexaenoic acid (DHA), play a role in the integrity of neuronal membranes, synaptic plasticity, and anti-inflammatory processes [85]. Epidemiologic studies have linked a higher dietary intake of DHA with a lower risk of cognitive decline and dementia [86]. Nevertheless, evidence from randomized controlled trials remains inconsistent, with some studies showing modest benefits and others reporting no significant effects on cognitive outcomes.

B vitamins, including B6, B12, and folate, are crucial for the metabolism of homocysteine, and deficiencies in these vitamins have been associated with higher levels of homocysteine, which constitute a risk factor for cognitive impairment and brain atrophy [87]. However, intervention studies aimed at lowering homocysteine levels through B-vitamin supplementation have yielded mixed results, and their impact on cognitive outcomes remains uncertain.

Vitamins E and C are antioxidants that help to combat oxidative stress, which is implicated in neuronal injury and cognitive decline [88]. Polyphenolic compounds, especially the flavonoids in green tea, cocoa, and berries, have been found to have the potential to induce neuroprotection via their anti-inflammatory and antioxidant activities [89]. Despite promising mechanistic evidence, clinical studies in humans are still limited and often characterized by short follow-up periods and heterogeneous outcomes.

As a precursor of the neurotransmitter acetylcholine, choline is important for cognitive performance, particularly memory and attention. Studies have linked dietary choline intake to better cognitive performance in older adults [90]. Despite the well-documented roles of these nutrients, most older adults fail to meet the recommended intake levels due to poor dietary habits, malabsorption, or the loss of appetite, thus highlighting the need for targeted nutritional interventions [91]. At the same time, differences in study populations, methods of cognitive assessment, and duration of follow-up limit the comparability of findings across studies and complicate the interpretation of results.

Overall, cognitive function represents a critical component of healthy aging, and nutritional factors may contribute to its preservation through multiple biological pathways, including the modulation of neuroinflammation, oxidative stress, and vascular health. However, current evidence remains heterogeneous and largely observational, thus limiting causal inference. Therefore, while dietary strategies may support cognitive health, their role in promoting healthy longevity requires further confirmation from well-designed long-term studies.

### 3.8. Physical Condition

#### 3.8.1. Background

Sustaining optimal physical fitness is crucial in promoting healthy longevity, due to its significant influence on mobility, independence, and overall quality of life. The loss of muscle mass, strength, and endurance associated with the aging process contributes to the development of sarcopenia and frailty, thus increasing the risk of disability and chronic diseases [70]. Although consistent engagement in physical activity is key in preventing these adverse effects, adequate nutrition is also essential to optimize muscle function and physical performance [92]. Malnutrition and inadequate dietary intake can accelerate muscle deterioration, whereas a well-balanced diet abundant in essential nutrients sustains musculoskeletal health, metabolic effectiveness, and overall longevity [93,94].

#### 3.8.2. Key Nutrients and Nutrient Gaps

Several key nutrients have a significant influence on the maintenance of physical condition and on preventing age-related muscle deterioration in older adults. Protein intake is fundamental for muscle protein synthesis. Research indicates that older adults require a higher intake to counteract anabolic resistance [77]. Leucine, a branched-chain amino acid, emerges as a potent stimulator of muscle protein synthesis, underscoring its critical dietary relevance for aging individuals [95].

Furthermore, vitamin D assumes a crucial role in supporting muscle function and strength. Deficiencies in this nutrient have been correlated with an increased risk of falls, muscle weakness, and frailty in older adults [96]. The intake of omega-3 fatty acids, in particular eicosapentaenoic acid (EPA) and docosahexaenoic acid (DHA), has demonstrated beneficial effects on muscle quality and function, which is primarily attributed to their anti-inflammatory properties [97].

In addition to macronutrients, micronutrients such as magnesium, potassium, and calcium are indispensable for neuromuscular function and musculoskeletal integrity. Magnesium and potassium deficiencies can compromise muscle contraction and energy metabolism [98,99], while calcium is essential for both muscle contraction and bone health, which are closely associated in aging individuals [100]. Despite the established significance of these nutrients, many older adults fail to achieve the recommended intake due to diminished appetite, compromised nutritional absorption, or dietary restrictions. This highlights the need for targeted dietary interventions.

Taken together, the current literature suggests that physical condition represents a key determinant of healthy aging, as it directly influences mobility, independence, and resilience to age-related decline. Adequate nutrition may support the maintenance of muscle mass and function, particularly via sufficient protein intake, micronutrient adequacy, and anti-inflammatory dietary components. However, the strength of evidence varies across nutrients and outcomes, and much of the available data derives from observational studies or short-term interventions. In this context, dietary strategies aimed at preserving physical function may contribute to healthier aging trajectories, although further long-term and well-controlled studies are needed to clarify their impact on longevity.

### 3.9. The Role of the Gut Microbiota

In recent years, a growing body of evidence from both in vivo and human studies has demonstrated that the gut microbiota plays a crucial role in the maintenance of optimal health and the onset and progression of obesity and related diseases. However, the strength and consistency of this evidence vary across study designs and populations. This is due to the complex interactions among the gut microbiota, diet, and host. The composition of the gut microbiota influences digestion, absorption, and nutrient metabolism, including lipid and glucose metabolism [101], while gut dysbiosis, which is commonly observed in pathological conditions, can affect body weight and overall health by altering the production of metabolites derived from dietary components [102]. For instance, in type 2 diabetes, gut microbiota diversity and composition are altered, with a decreased abundance of beneficial bacteria such as *Faecalibacterium* and *Bifidobacterium*, but there are antidiabetic treatments that may partially restore these populations and contribute to improved glucose control [103]. Dysbiosis also plays a role in hypertension through inflammatory, metabolic, and neural mechanisms, highlighting the potential of dietary and microbial interventions in blood pressure management [104]. Nevertheless, most human evidence remains observational, and causal relationships are often difficult to disentangle from confounding dietary and lifestyle factors. Although several mechanisms such as inflammatory, hormonal, and metabolic pathways have been proposed, a clear understanding of these interactions remains elusive [105,106].

Emerging evidence also suggests a bidirectional relationship between microbiota and sleep quality, as disruptions in circadian rhythms can lead to microbial imbalance, which in turn exacerbates sleep disturbances. Dietary factors such as fiber, unsaturated fatty acids, and bioactive compounds like polyphenols, along with meal timing, modulate microbial metabolite production and influence sleep through pathways involving GABA, serotonin, and melatonin [27,107,108]. The gut microbiota also impacts physical performance and muscle recovery by modulating energy metabolism, inflammation, and immune responses, whereas dysbiosis induced by poor diet and inactivity can impair muscle function [109,110,111]. Similarly, the gut–brain axis plays a key role in cognitive health, with microbiota composition influencing neuroinflammation, neurotransmitter synthesis, and cognitive performance, and dysbiosis has thus been linked to neurodegenerative diseases such as Alzheimer’s and Parkinson’s [112,113,114,115]. However, inter-individual variability, methodological heterogeneity, and sparse longitudinal data currently limit the clinical translation of these findings. Finally, the gut–muscle axis further underscores the role of microbiota in systemic inflammation and muscle catabolism, with dietary interventions rich in fiber, probiotics, and fermented foods emerging as promising strategies to support metabolic, cognitive, and physical health [115,116].

Overall, the gut microbiota may represent an important interface linking dietary exposures to multiple biological processes relevant to aging, including metabolic regulation, inflammation, and neurocognitive function. However, given the predominance of observational evidence and the high inter-individual variability, its role in promoting healthy longevity remains to be fully elucidated. In this context, the dietary modulation of the microbiota may contribute to healthier aging trajectories, although the extent and clinical relevance of these effects require further investigation.

**Figure 2:**
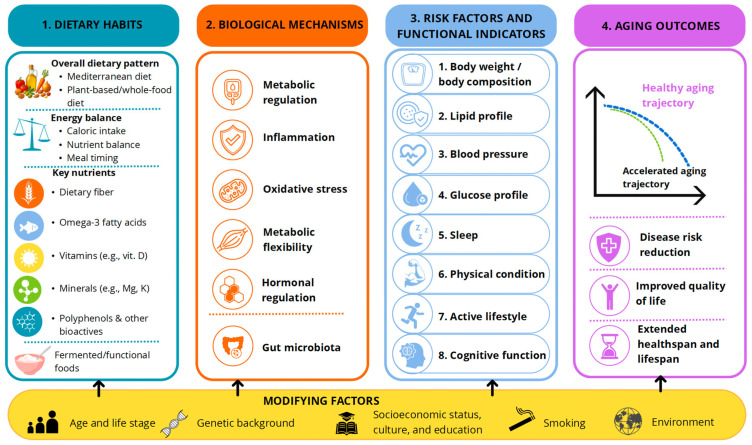
Conceptual framework summarizing relationships between dietary factors, biological pathways, and determinants of healthy aging.

Figure 2 synthesizes the main elements discussed in the previous sections, highlighting the interactions between dietary exposures, biological pathways, and determinants relevant to healthy aging trajectories.

## 4. Conclusions

To promote healthy longevity, a comprehensive approach that combines balanced nutrition with an active lifestyle is essential. Key dietary recommendations may include increasing the intake of fiber-rich foods, prebiotics, probiotics, and omega-3 fatty acids from sources such as fatty fish, nuts, and seeds. The diet should also provide an appropriate energy intake to support physiological needs and maintain a healthy body weight over time. Maintaining long-term energy balance is considered a central factor in preventing excess adiposity and metabolic dysfunction. Besides this aspect, high-quality protein, ideally distributed throughout the day, and essential micronutrients such as vitamin D, B-group vitamins (particularly B6 and B12), magnesium, zinc, and selenium may play an important role, especially for at-risk populations (e.g., individuals with diabetes or hypercholesterolemia or older adults). Improving fat quality, limiting sodium, and incorporating targeted supplementation may be considered in populations at risk of deficiency. Fermented foods and plant-based dietary patterns have been associated with the beneficial modulation of the gut microbiota, which, in turn, supports metabolic, cardiovascular, cognitive, and muscular health, although the magnitude of these effects appears to vary between individuals. Additionally, the regular consumption of antioxidant-rich foods may contribute to counteracting oxidative stress and supporting overall resilience. Adequate hydration is also likely to play an important role, as even mild dehydration can impair physical and cognitive performance. Regular physical activity, including both aerobic and resistance training, is important for preserving muscle mass, functional mobility, and brain function. Finally, behavioral strategies such as minimizing sedentary time, engaging in social and outdoor activities, ensuring sufficient and restorative sleep, and maintaining energy balance are all fundamental to achieving healthy longevity. Future research should better integrate traditional clinical risk factors with emerging molecular biomarkers of biological aging to clarify the extent to which nutritional and lifestyle interventions may [25] influence biological aging processes instead of solely improving disease risk profiles.

## Data Availability

The original contributions presented in this study are included in the article. Further inquiries can be directed to the corresponding author.

## References

[1] Hay S.I., Ong K.L., Santomauro D.F., Bhoomadevi A., Aalipour M.A., Aalruz H., Ababneh H.S., Abaraogu U.O., Abate B.B., Abbafati C. (2025). Burden of 375 Diseases and Injuries, Risk-Attributable Burden of 88 Risk Factors, and Healthy Life Expectancy in 204 Countries and Territories, Including 660 Subnational Locations, 1990–2023: A Systematic Analysis for the Global Burden of Disease Study 2023. Lancet.

[2] Afshin A., Sur P.J., Fay K.A., Cornaby L., Ferrara G., Salama J.S., Mullany E.C., Abate K.H., Abbafati C., Abebe Z. (2019). Health Effects of Dietary Risks in 195 Countries, 1990–2017: A Systematic Analysis for the Global Burden of Disease Study 2017. Lancet.

[3] Phelps N.H., Singleton R.K., Zhou B., Heap R.A., Mishra A., Bennett J.E., Paciorek C.J., Lhoste V.P., Carrillo-Larco R.M., Stevens G.A. (2024). Worldwide Trends in Underweight and Obesity from 1990 to 2022: A Pooled Analysis of 3663 Population-Representative Studies with 222 Million Children, Adolescents, and Adults. Lancet.

[4] Zhou X.D., Chen Q.F., Yang W., Zuluaga M., Targher G., Byrne C.D., Valenti L., Luo F., Katsouras C.S., Thaher O. (2024). Burden of Disease Attributable to High Body Mass Index: An Analysis of Data from the Global Burden of Disease Study 2021. EClinicalMedicine.

[5] Ferrari A.J., Santomauro D.F., Aali A., Abate Y.H., Abbafati C., Abbastabar H., ElHafeez S.A., Abdelmasseh M., Abd-Elsalam S., Abdollahi A. (2024). Global Incidence, Prevalence, Years Lived with Disability (YLDs), Disability-Adjusted Life-Years (DALYs), and Healthy Life Expectancy (HALE) for 371 Diseases and Injuries in 204 Countries and Territories and 811 Subnational Locations, 1990–2021: A System Analysis for the Global Burden of Disease Study 2021. Lancet.

[6] Figlioli G., Piovani D., Tsantes A.G., Pugliese N., Nikolopoulos G.K., Hassan C., Repici A., Lleo A., Aghemo A., Bonovas S. (2025). Burden of Cancer Attributable to High Body Mass Index: A Systematic Analysis of the Global Burden of Disease Study 2021. Clin. Nutr..

[7] THE 17 GOALS|Sustainable Development. https://sdgs.un.org/goals.

[8] Dinu M., Pagliai G., Angelino D., Rosi A., Dall’Asta M., Bresciani L., Ferraris C., Guglielmetti M., Godos J., Del Bo C. (2020). Effects of Popular Diets on Anthropometric and Cardiometabolic Parameters: An Umbrella Review of Meta-Analyses of Randomized Controlled Trials. Adv. Nutr..

[9] Tessier A.J., Wang F., Korat A.A., Eliassen A.H., Chavarro J., Grodstein F., Li J., Liang L., Willett W.C., Sun Q. (2025). Optimal Dietary Patterns for Healthy Aging. Nat. Med..

[10] Dinu M., Martini D., Sofi F., Serafini M., Porrini M., Angelino D. (2023). Comparison of Modern-Millennial Diets. Handbook of Obesity-Volume 2: Clinical Applications.

[11] Klos B., Cook J., Crepaz L., Weiland A., Zipfel S., Mack I. (2023). Impact of Energy Density on Energy Intake in Children and Adults: A Systematic Review and Meta-Analysis of Randomized Controlled Trials. Eur. J. Nutr..

[12] Calvani R., Picca A., Coelho-Júnior H.J., Tosato M., Marzetti E., Landi F. (2023). Diet for the Prevention and Management of Sarcopenia. Metabolism.

[13] Dramé M., Godaert L. (2023). The Obesity Paradox and Mortality in Older Adults: A Systematic Review. Nutrients.

[14] Patel P.N., Giugliano R.P. (2020). Low-Density Lipoprotein Cholesterol Lowering Therapy for the Secondary Prevention of Atherosclerotic Cardiovascular Disease. Glob. Cardiol. Sci. Pract..

[15] Jenkins D.J.A., Kendall C.W.C., Marchie A., Faulkner D.A., Wong J.M.W., De Souza R., Emam A., Parker T.L., Vidgen E., Trautwein E.A. (2005). Direct Comparison of a Dietary Portfolio of Cholesterol-Lowering Foods with a Statin in Hypercholesterolemic Participants. Am. J. Clin. Nutr..

[16] Maștaleru A., Cojocariu A.S., Oancea A., Leon-Constantin M.M., Roca M., Zota I.M., Abdulan I.M., Rusu C., Trandafir L.M., Costache A.D. (2022). Eating Habits in Patients with Familial Hypercholesterolemia from North-Eastern Romania. Nutrients.

[17] Na L., Han T., Zhang W., Wu X., Na G., Du S., Li Y., Sun C. (2015). A Snack Dietary Pattern Increases the Risk of Hypercholesterolemia in Northern Chinese Adults: A Prospective Cohort Study. PLoS ONE.

[18] Gora A.H., Rehman S., Kiron V., Dias J., Fernandes J.M.O., Olsvik P.A., Siriyappagouder P., Vatsos I., Schmid-Staiger U., Frick K. (2022). Management of Hypercholesterolemia Through Dietary SS-Glucans–Insights From a Zebrafish Model. Front. Nutr..

[19] Popiolek-Kalisz J., Salamon K., Mazur M., Mikolajczyk K., Kalisz G. (2025). Dietary Approach in Familial Hypercholesterolemia. Cardiogenetics.

[20] Zhang C., Wei G., Zhou H., Liu L. (2024). Causal Relationships of Familial Hypercholesterolemia with the Risk of Multiple Vitamin Deficiencies: A Mendelian Randomization Study. Front. Endocrinol..

[21] Krishnamurthy H.K., Reddy S., Jayaraman V., Krishna K., Song Q., Wang T., Bei K., Rajasekaran J.J. (2023). Profiling the Effect of Micronutrient Levels on Vital Cardiac Markers. medRxiv.

[22] Al-Qusous M.N., Al Madanat W.K.J., Hussein R.M. (2023). Association of Vitamins D, B6, and B12 Deficiencies with Hyperlipidemia Among Jordanian Adults. Rep. Biochem. Mol. Biol..

[23] Freitas De Carvalho L.M., Batista Beserra J., De Sousa Carvalho L., De Sousa C.B., Sampaio Da Paz S.M.R., Dos Santos M.M. (2020). Association between Magnesium, Selenium and Zinc Consumption and Lipid Profile of Brazilian Adolescents. Rev. Chil. De Nutr..

[24] Teymoori F., Asghari G., Salehi P., Sadeghian S., Mirmiran P., Azizi F. (2019). Are dietary amino acids prospectively predicts changes in serum lipid profile?. Diabetes Metab. Syndr..

[25] McGarrah R.W., White P.J. (2023). Branched-Chain Amino Acids in Cardiovascular Disease. Nat. Rev. Cardiol..

[26] Kianmehr H., Zhang P., Luo J., Guo J., Pavkov M.E., Bullard K.M.K., Gregg E.W., Ospina N.S., Fonseca V., Shi L. (2022). Potential Gains in Life Expectancy Associated With Achieving Treatment Goals in US Adults With Type 2 Diabetes. JAMA Netw. Open.

[27] Lin Z., Jiang T., Chen M., Ji X., Wang Y. (2024). Gut Microbiota and Sleep: Interaction Mechanisms and Therapeutic Prospects. Open Life Sci..

[28] Bross R., Genter P., Lu Y., Serpas L., Campa D., Ipp E. (2022). Barriers to Healthy Eating and Diabetes Diet Education: Divergent Perspectives of Patients and Their Providers. Health Educ. Behav..

[29] Landa-Anell M.V., Melgarejo-Hernández M.A., García-Ulloa A.C., Del Razo-Olvera F.M., Velázquez-Jurado H.R., Hernández-Jiménez S. (2020). Barriers to Adherence to a Nutritional Plan and Strategies to Overcome Them in Patients with Type 2 Diabetes Mellitus; Results after Two Years of Follow-Up. Endocrinol. Diabetes Nutr..

[30] Mangal D.K., Shaikh N., Tolani H., Gautam D., Pandey A.K., Sonnathi Y., Gupta S.D., Kalra S., Sharma K.C., Prasad J. (2025). Burden of Micronutrient Deficiency among Patients with Type 2 Diabetes: Systematic Review and Meta-Analysis. BMJ Nutr. Prev. Health.

[31] Walker A.F. (2007). Potential Micronutrient Deficiency Lacks Recognition in Diabetes. Br. J. Gen. Pract..

[32] Luo B., Pan B., Zhao G., Li J., Sun L. (2024). Association Between Serum Magnesium Levels and Glycemic Control in Type 2 Diabetes. Diabetes Metab. Syndr. Obes..

[33] Xu L., Li X., Wang X., Xu M. (2023). Effects of Magnesium Supplementation on Improving Hyperglycemia, Hypercholesterolemia, and Hypertension in Type 2 Diabetes: A Pooled Analysis of 24 Randomized Controlled Trials. Front. Nutr..

[34] Norouzi S., Adulcikas J., Sohal S.S., Myers S. (2018). Zinc Stimulates Glucose Oxidation and Glycemic Control by Modulating the Insulin Signaling Pathway in Human and Mouse Skeletal Muscle Cell Lines. PLoS ONE.

[35] Yoon J.-S. (2008). Zinc Status and Dietary Quality of Type 2 Diabetic Patients: Implication of Physical Activity Level. Nutr. Res. Pract..

[36] Farooq D., Alamri A., Alwhahabi B., Metwally A., Kareem K. (2020). The Status of Zinc in Type 2 Diabetic Patients and Its Association with Glycemic Control. J. Fam. Community Med..

[37] Swidan A.K., Ahmed M.A.S. (2023). Should We Follow the Guidelines on Vitamin B12 Deficiency and Diabetes? A Retrospective Analysis of Data from Middle Eastern Population. Alex. J. Med..

[38] Kibirige D., Mwebaze R. (2013). Vitamin B12 Deficiency among Patients with Diabetes Mellitus: Is Routine Screening and Supplementation Justified? *J*. Diabetes Metab. Disord..

[39] Neal E.S., Kumar V., Borges K., Cuffe J.S.M. (2023). Vitamin B12 Deficiency Induces Glucose Intolerance, Delays Peak Insulin Levels and Promotes Ketogenesis in Female Rats. J. Endocrinol..

[40] Niroomand M., Fotouhi A., Irannejad N., Hosseinpanah F. (2019). Does High-Dose Vitamin D Supplementation Impact Insulin Resistance and Risk of Development of Diabetes in Patients with Pre-Diabetes? A Double-Blind Randomized Clinical Trial. Diabetes Res. Clin. Pract..

[41] Barbarawi M., Zayed Y., Barbarawi O., Bala A., Alabdouh A., Gakhal I., Rizk F., Alkasasbeh M., Bachuwa G., Manson J.E. (2020). Effect of Vitamin D Supplementation on the Incidence of Diabetes Mellitus. J. Clin. Endocrinol. Metab..

[42] Zhang Y., Tan H., Tang J., Li J., Chong W., Hai Y., Feng Y., Lunsford L.D., Xu P., Jia D. (2020). Effects of Vitamin D Supplementation on Prevention of Type 2 Diabetes in Patients With Prediabetes: A Systematic Review and Meta-Analysis. Diabetes Care.

[43] Pittas A.G., Jorde R., Kawahara T., Dawson-Hughes B. (2020). Vitamin D Supplementation for Prevention of Type 2 Diabetes Mellitus: To D or Not to D? *J*. Clin. Endocrinol. Metab..

[44] Demay M.B., Pittas A.G., Bikle D.D., Diab D.L., Kiely M.E., Lazaretti-Castro M., Lips P., Mitchell D.M., Murad M.H., Powers S. (2024). Vitamin D for the Prevention of Disease: An Endocrine Society Clinical Practice Guideline. J. Clin. Endocrinol. Metab..

[45] George K.M., Maillard P., Gilsanz P., Fletcher E., Peterson R.L., Fong J., Mayeda E.R., Mungas D.M., Barnes L.L., Glymour M.M. (2023). Association of Early Adulthood Hypertension and Blood Pressure Change With Late-Life Neuroimaging Biomarkers. JAMA Netw. Open.

[46] Vignesh A., Amal T.C., Shanmugam A., Vasanth K., Selvakumar S. (2025). Effects of Dietary Approaches to Prevent Hypertension and Enhance Cardiovascular Health. Discov. Food.

[47] Houston M.C., Harper K.J. (2008). Potassium, Magnesium, and Calcium: Their Role in Both the Cause and Treatment of Hypertension. J. Clin. Hypertens..

[48] Altawili A.A., Altawili M., Alwadai A.M., Alahmadi A.S., Alshehri A.M.A., Muyini B.H., Alshwwaf A.R., Almarzooq A.M., Alqarni A.H.A., Alruwili Z.A.L. (2023). An Exploration of Dietary Strategies for Hypertension Management: A Narrative Review. Cureus.

[49] Nguyen H., Odelola O.A., Rangaswami J., Amanullah A. (2013). A Review of Nutritional Factors in Hypertension Management. Int. J. Hypertens..

[50] The Effectiveness of Salt Restriction Versus Other Non-Pharmacological Approaches to Prevent or Control Arterial Hypertension. https://www.escardio.org/Journals/E-Journal-of-Cardiology-Practice/Volume-22/the-effectiveness-of-salt-restriction-versus-other-non-pharmacological-approache.

[51] Wasilewski A., Marczyński P., Kontek S., Jabłoński F., Kasprzak A., Wasilewska E., Kosendiak A.A. (2024). Nutritional Discrepancies Among Inpatients and Outpatients Diagnosed with Hypertension. Healthcare.

[52] Sella E., Miola L., Toffalini E., Borella E. (2023). The Relationship between Sleep Quality and Quality of Life in Aging: A Systematic Review and Meta-Analysis. Health Psychol. Rev..

[53] Yazdi Z., Sadeghniiat-Haghighi K., Loukzadeh Z., Elmizadeh K., Abbasi M. (2014). Prevalence of Sleep Disorders and Their Impacts on Occupational Performance: A Comparison between Shift Workers and Nonshift Workers. Sleep Disord..

[54] Musshafen L.A., Tyrone R.S., Abdelaziz A., Sims-Gomillia C.E., Pongetti L.S., Teng F., Fletcher L.M., Reneker J.C. (2021). Associations between Sleep and Academic Performance in US Adolescents: A Systematic Review and Meta-Analysis. Sleep Med..

[55] Kwok C.S., Kontopantelis E., Kuligowski G., Gray M., Muhyaldeen A., Gale C.P., Peat G.M., Cleator J., Chew-Graham C., Loke Y.K. (2018). Self-Reported Sleep Duration and Quality and Cardiovascular Disease and Mortality: A Dose-Response Meta-Analysis. J. Am. Heart Assoc..

[56] Bacaro V., Ballesio A., Cerolini S., Vacca M., Poggiogalle E., Donini L.M., Lucidi F., Lombardo C. (2020). Sleep Duration and Obesity in Adulthood: An Updated Systematic Review and Meta-Analysis. Obes. Res. Clin. Pract..

[57] Matricciani L., Bin Y.S., Lallukka T., Kronholm E., Wake M., Paquet C., Dumuid D., Olds T. (2018). Rethinking the Sleep-Health Link. Sleep Health.

[58] Gradisar M., Gardner G., Dohnt H. (2011). Recent Worldwide Sleep Patterns and Problems during Adolescence: A Review and Meta-Analysis of Age, Region, and Sleep. Sleep Med..

[59] Brito R.S., Dias C., Filho A.A., Salles C. (2021). Prevalence of Insomnia in Shift Workers: A Systematic Review. Sleep Sci..

[60] Canever J.B., Zurman G., Vogel F., Sutil D.V., Diz J.B.M., Danielewicz A.L., Moreira B.D.S., Cimarosti H.I., de Avelar N.C.P. (2024). Worldwide Prevalence of Sleep Problems in Community-Dwelling Older Adults: A Systematic Review and Meta-Analysis. Sleep Med..

[61] Dashti H.S., Scheer F.A.J.L., Jacques P.F., Lamon-Fava S., Ordovás J.M. (2015). Short Sleep Duration and Dietary Intake: Epidemiologic Evidence, Mechanisms, and Health Implications. Adv. Nutr..

[62] Lundahl A., Nelson T.D. (2015). Sleep and Food Intake: A Multisystem Review of Mechanisms in Children and Adults. J. Health Psychol..

[63] Godos J., Ferri R., Lanza G., Caraci F., Vistorte A.O.R., Yelamos Torres V., Grosso G., Castellano S. (2024). Mediterranean Diet and Sleep Features: A Systematic Review of Current Evidence. Nutrients.

[64] St-Onge M.P., Mikic A., Pietrolungo C.E. (2016). Effects of Diet on Sleep Quality. Adv. Nutr..

[65] Wilson K., St-Onge M.P., Tasali E. (2022). Diet Composition and Objectively Assessed Sleep Quality: A Narrative Review. J. Acad. Nutr. Diet..

[66] EFSA (2017). Dietary Reference Values for Nutrients Summary Report. EFSA Support. Public..

[67] Booth F.W., Roberts C.K., Laye M.J. (2012). Lack of Exercise Is a Major Cause of Chronic Diseases. Compr. Physiol..

[68] Sánchez-Sánchez J.L., Ortolá R., Banegas J.R., Lucia A., Rodríguez-Artalejo F., Sotos-Prieto M., Valenzuela P.L. (2025). Association between Physical Activity and Sedentary Behaviour and Changes in Intrinsic Capacity in Spanish Older Adults (Seniors-ENRICA-2): A Prospective Population-Based Study. Lancet Healthy Longev..

[69] Cacciatore S., Calvani R., Prokopidis K., Schlögl M., Russo A., Tosato M., Anton S.D., Leeuwenburgh C., Batsis J.A., Marzetti E. (2026). Intrinsic Capacity–Frailty Phenotypes and Subclinical Inflammation in Community-Dwelling Octogenarians: A Cross-Sectional Analysis from the IlSIRENTE Study. Exp. Gerontol..

[70] Cruz-Jentoft A.J., Bahat G., Bauer J., Boirie Y., Bruyère O., Cederholm T., Cooper C., Landi F., Rolland Y., Sayer A.A. (2019). Sarcopenia: Revised European Consensus on Definition and Diagnosis. Age Ageing.

[71] Pedersen B.K. (2019). Physical Activity and Muscle–Brain Crosstalk. Nat. Rev. Endocrinol..

[72] Borda M.G., Landi F., Cederholm T., Venegas-Sanabria L.C., Duque G., Wakabayashi H., Barreto G.E., Rodriguez-Sanchez I., Canevelli M., Cano-Gutierrez C. (2025). Assessment and Management of Frailty in Individuals Living with Dementia: Expert Recommendations for Clinical Practice. Lancet Healthy Longev..

[73] Ekelund U., Tarp J., Fagerland M.W., Johannessen J.S., Hansen B.H., Jefferis B.J., Whincup P.H., Diaz K.M., Hooker S., Howard V.J. (2020). Joint Associations of Accelero-Meter Measured Physical Activity and Sedentary Time with All-Cause Mortality: A Harmonised Meta-Analysis in More than 44 000 Middle-Aged and Older Individuals. Br. J. Sports Med..

[74] Bernabei R., Landi F., Calvani R., Cesari M., Del Signore S., Anker S.D., Bejuit R., Bordes P., Cherubini A., Cruz-Jentoft A.J. (2022). Multicomponent Intervention to Prevent Mobility Disability in Frail Older Adults: Randomised Controlled Trial (SPRINTT Project). BMJ.

[75] Qaisar R., Hussain M.A., Naheed S., Saeed K., Karim A., Ahmad F., Haider S., Alhussain M.H., Alkahtani S.A. (2026). Low Protein Intake Is Associated with the Risk of Functional Impairment in Older Adults in an Age- and Gender-Specific Manner: A SHARE-Based Study. Nutrients.

[76] Devries M.C., Phillips S.M. (2015). Supplemental Protein in Support of Muscle Mass and Health: Advantage Whey. J. Food Sci..

[77] Bauer J., Biolo G., Cederholm T., Cesari M., Cruz-Jentoft A.J., Morley J.E., Phillips S., Sieber C., Stehle P., Teta D. (2013). Evidence-Based Recommendations for Optimal Dietary Protein Intake in Older People: A Position Paper from the Prot-Age Study Group. J. Am. Med. Dir. Assoc..

[78] Slavin J.L. (2005). Dietary Fiber and Body Weight. Nutrition.

[79] Maughan R.J. (2012). Hydration, Morbidity, and Mortality in Vulnerable Populations. Nutr. Rev..

[80] Bischoff-Ferrari H.A. (2011). Relevance of Vitamin D in Muscle Health. Rev. Endocr. Metab. Disord..

[81] Philpott J.D., Donnelly C., Walshe I.H., MacKinley E.E., Dick J., Galloway S.D.R., Tipton K.D., Witard O.C. (2018). Adding Fish Oil to Whey Protein, Leucine, and Carbohydrate Over a Six-Week Supplementation Period Attenuates Muscle Soreness Following Eccentric Exercise in Competitive Soccer Players. Int. J. Sport Nutr. Exerc. Metab..

[82] Livingston G., Huntley J., Sommerlad A., Ames D., Ballard C., Banerjee S., Brayne C., Burns A., Cohen-Mansfield J., Cooper C. (2020). Dementia Prevention, Intervention, and Care: 2020 Report of the Lancet Commission. Lancet.

[83] Jacka F.N., Cherbuin N., Anstey K.J., Sachdev P., Butterworth P. (2015). Western Diet Is Associated with a Smaller Hippocampus: A Longitudinal Investigation. BMC Med..

[84] Livingston G., Huntley J., Liu K.Y., Costafreda S.G., Selbæk G., Alladi S., Ames D., Banerjee S., Burns A., Brayne C. (2024). Dementia Prevention, Intervention, and Care: 2024 Report of the Lancet Standing Commission. Lancet.

[85] Cunnane S.C., Plourde M., Pifferi F., Bégin M., Féart C., Barberger-Gateau P. (2009). Fish, Docosahexaenoic Acid and Alzheimer’s Disease. Prog. Lipid Res..

[86] Yurko-Mauro K., McCarthy D., Rom D., Nelson E.B., Ryan A.S., Blackwell A., Salem N., Stedman M. (2010). Beneficial Effects of Docosahexaenoic Acid on Cognition in Age-Related Cognitive Decline. Alzheimers Dement..

[87] Smith A.D., Refsum H. (2016). Homocysteine, B Vitamins, and Cognitive Impairment. Annu. Rev. Nutr..

[88] Joseph J., Cole G., Head E., Ingram D. (2009). Nutrition, Brain Aging, and Neurodegeneration. J. Neurosci..

[89] Spencer J.P.E., Vauzour D., Rendeiro C. (2009). Flavonoids and Cognition: The Molecular Mechanisms Underlying Their Behavioural Effects. Arch. Biochem. Biophys..

[90] Nurk E., Refsum H., Drevon C.A., Tell G.S., Nygaard H.A., Engedal K., Smith A.D. (2009). Intake of Flavonoid-Rich Wine, Tea, and Chocolate by Elderly Men and Women Is Associated with Better Cognitive Test Performance. J. Nutr..

[91] Morris M.C., Tangney C.C., Wang Y., Sacks F.M., Bennett D.A., Aggarwal N.T. (2015). MIND Diet Associated with Reduced Incidence of Alzheimer’s Disease. Alzheimers Dement..

[92] Cacciatore S., Calvani R., Esposito I., Massaro C., Gava G., Picca A., Tosato M., Marzetti E., Landi F. (2024). Emerging Targets and Treatments for Sarcopenia: A Narrative Review. Nutrients.

[93] Deutz N.E.P., Bauer J.M., Barazzoni R., Biolo G., Boirie Y., Bosy-Westphal A., Cederholm T., Cruz-Jentoft A., Krznariç Z., Nair K.S. (2014). Protein Intake and Exercise for Optimal Muscle Function with Aging: Recommendations from the ESPEN Expert Group. Clin. Nutr..

[94] Cacciatore S., Calvani R., Marzetti E., Picca A., Coelho-Júnior H.J., Martone A.M., Massaro C., Tosato M., Landi F. (2023). Low Adherence to Mediterranean Diet Is Associated with Probable Sarcopenia in Community-Dwelling Older Adults: Results from the Longevity Check-Up (Lookup) 7+ Project. Nutrients.

[95] Katsanos C.S., Kobayashi H., Sheffield-Moore M., Aarsland A., Wolfe R.R. (2006). A High Proportion of Leucine Is Required for Optimal Stimulation of the Rate of Muscle Protein Synthesis by Essential Amino Acids in the Elderly. Am. J. Physiol. Endocrinol. Metab..

[96] Bischoff-Ferrari H.A., Dietrich T., Orav E.J., Dawson-Hughes B. (2004). Positive Association between 25-Hydroxy Vitamin D Levels and Bone Mineral Density: A Population-Based Study of Younger and Older Adults. Am. J. Med..

[97] Smith G.I., Julliand S., Reeds D.N., Sinacore D.R., Klein S., Mittendorfer B. (2015). Fish Oil-Derived n-3 PUFA Therapy Increases Muscle Mass and Function in Healthy Older Adults. Am. J. Clin. Nutr..

[98] Volpe S.L. (2013). Magnesium in Disease Prevention and Overall Health. Adv. Nutr..

[99] Yamada S., Inaba M. (2021). Potassium Metabolism and Management in Patients with CKD. Nutrients.

[100] Bendahan D., Mattei J.P., Ghattas B., Confort-Gouny S., Le Guern M.E., Cozzone P.J. (2002). Citrulline/Malate Promotes Aerobic Energy Production in Human Exercising Muscle. Br. J. Sports Med..

[101] Rowland I., Gibson G., Heinken A., Scott K., Swann J., Thiele I., Tuohy K. (2018). Gut Microbiota Functions: Metabolism of Nutrients and Other Food Components. Eur. J. Nutr..

[102] Perler B.K., Friedman E.S., Wu G.D. (2023). The Role of the Gut Microbiota in the Relationship Between Diet and Human Health. Annu. Rev. Physiol..

[103] Almugadam B.S., Liu Y., Chen S.M., Wang C.H., Shao C.Y., Ren B.W., Tang L. (2020). Alterations of Gut Microbiota in Type 2 Diabetes Individuals and the Confounding Effect of Antidiabetic Agents. J. Diabetes Res..

[104] Młynarska E., Wasiak J., Gajewska A., Bilińska A., Steć G., Jasińska J., Rysz J., Franczyk B. (2024). Gut Microbiota and Gut-Brain Axis in Hypertension: Implications for Kidney and Cardiovascular Health-A Narrative Review. Nutrients.

[105] Cuevas-Sierra A., Ramos-Lopez O., Riezu-Boj J.I., Milagro F.I., Martinez J.A. (2019). Diet, Gut Microbiota, and Obesity: Links with Host Genetics and Epigenetics and Potential Applications. Adv. Nutr..

[106] Geng J., Ni Q., Sun W., Li L., Feng X. (2022). The Links between Gut Microbiota and Obesity and Obesity Related Diseases. Biomed. Pharmacother..

[107] Yang D.F., Huang W.C., Wu C.W., Huang C.Y., Yang Y.C.S.H., Tung Y.T. (2023). Acute Sleep Deprivation Exacerbates Systemic Inflammation and Psychiatry Disorders through Gut Microbiota Dysbiosis and Disruption of Circadian Rhythms. Microbiol. Res..

[108] Sejbuk M., Siebieszuk A., Witkowska A.M. (2024). The Role of Gut Microbiome in Sleep Quality and Health: Dietary Strategies for Microbiota Support. Nutrients.

[109] Clarke S.F., Murphy E.F., O’Sullivan O., Lucey A.J., Humphreys M., Hogan A., Hayes P., O’Reilly M., Jeffery I.B., Wood-Martin R. (2014). Exercise and Associated Dietary Extremes Impact on Gut Microbial Diversity. Gut.

[110] Cerdá B., Pérez M., Pérez-Santiago J.D., Tornero-Aguilera J.F., González-Soltero R., Larrosa M. (2016). Gut Microbiota Modification: Another Piece in the Puzzle of the Benefits of Physical Exercise in Health? *Front*. Physiol..

[111] Jäger R., Mohr A.E., Carpenter K.C., Kerksick C.M., Purpura M., Moussa A., Townsend J.R., Lamprecht M., West N.P., Black K. (2019). International Society of Sports Nutrition Position Stand: Probiotics. J. Int. Soc. Sports Nutr..

[112] Cryan J.F., O’riordan K.J., Cowan C.S.M., Sandhu K.V., Bastiaanssen T.F.S., Boehme M., Codagnone M.G., Cussotto S., Fulling C., Golubeva A.V. (2019). The Microbiota-Gut-Brain Axis. Physiol. Rev..

[113] Kowalski K., Mulak A. (2019). Brain-Gut-Microbiota Axis in Alzheimer’s Disease. J. Neurogastroenterol. Motil..

[114] Silva Y.P., Bernardi A., Frozza R.L. (2020). The Role of Short-Chain Fatty Acids From Gut Microbiota in Gut-Brain Communication. Front. Endocrinol..

[115] Ticinesi A., Lauretani F., Milani C., Nouvenne A., Tana C., Del Rio D., Maggio M., Ventura M., Meschi T. (2017). Aging Gut Microbiota at the Cross-Road between Nutrition, Physical Frailty, and Sarcopenia: Is There a Gut-Muscle Axis?. Nutrients.

[116] Ghosh T.S., Rampelli S., Jeffery I.B., Santoro A., Neto M., Capri M., Giampieri E., Jennings A., Candela M., Turroni S. (2020). Mediterranean Diet Intervention Alters the Gut Microbiome in Older People Reducing Frailty and Improving Health Status: The NU-AGE 1-Year Dietary Intervention across Five European Countries. Gut.

